# Systemic Citrate Toxicity During Regional Citrate Anticoagulation for Continuous Kidney Replacement Therapy

**DOI:** 10.3390/jcm15176564

**Published:** 2026-08-25

**Authors:** Sebastien Redant, Rachid Attou, Mircea T. Talpos, Patrick M. Honoré

**Affiliations:** 1 Department of Intensive Care, Brugmann University Hospital, Université Libre de Bruxelles, Place Van Gehuchtenplein 4, 1020 Brussels, Belgium; rachid.attou@chu-brugmann.be (R.A.); mirceatamas.talpos@chu-brugmann.be (M.T.T.); 2 Department of Intensive Care, CHU UCL Godinne Namur, UCLouvain Medical School, 5530 Yvoir, Belgium; patrick.honore@chuuclnamur.uclouvain.be

**Keywords:** regional citrate anticoagulation, continuous kidney replacement therapy, citrate accumulation, citrate toxicity, circulatory shock, mitochondrial metabolism

## Abstract

Regional citrate anticoagulation (RCA) is the preferred anticoagulation strategy for continuous kidney replacement therapy (CKRT) because it prolongs circuit lifespan while reducing bleeding complications compared with systemic heparin. Although generally safe, systemic citrate accumulation remains the principal metabolic complication of RCA and occurs when citrate delivery exceeds the combined metabolic and extracorporeal capacity for citrate elimination. This narrative review summarizes current evidence regarding citrate metabolism, the pathophysiological mechanisms of citrate accumulation, associated risk factors, biochemical diagnosis, monitoring strategies, and therapeutic management. Citrate metabolism depends predominantly on mitochondrial oxidative capacity rather than hepatic function alone, making circulatory shock and impaired tissue perfusion the principal determinants of citrate intolerance, whereas isolated liver dysfunction is often well tolerated. Systemic citrate accumulation is characterized by a distinctive biochemical profile including ionized hypocalcemia, an increased total-to-ionized calcium ratio, escalating calcium infusion requirements, high anion-gap metabolic acidosis, and frequently hyperlactatemia, while overt clinical manifestations usually occur late. Consequently, early recognition relies on structured biochemical monitoring and interpretation of serial trends rather than isolated measurements. Management should focus on restoring the balance between citrate delivery and citrate elimination by reducing citrate exposure when appropriate, optimizing extracorporeal citrate removal, correcting metabolic abnormalities, and improving tissue perfusion. Contemporary RCA practice should therefore emphasize individualized physiological assessment and integrated biochemical monitoring to facilitate early recognition, prevent progression to overt toxicity, and preserve the well-established safety and efficacy of RCA.

## 1. Introduction

Regional citrate anticoagulation (RCA) is the preferred anticoagulation strategy for continuous kidney replacement therapy (CKRT) because it prolongs circuit lifespan while reducing bleeding complications compared with systemic unfractionated heparin, particularly in patients at high risk of hemorrhage [1,2,3,4,5,6]. Citrate achieves regional anticoagulation by chelating ionized calcium, an essential cofactor in multiple steps of the coagulation cascade, thereby preventing clot formation within the extracorporeal circuit while largely preserving systemic coagulation.

Over the past decade, experimental and clinical studies have substantially refined the understanding of citrate metabolism and systemic handling during RCA. Rather than representing a consequence of isolated hepatic dysfunction, systemic citrate accumulation is now recognized as a mismatch between citrate delivery and the combined metabolic and extracorporeal capacity for citrate elimination [7,8,9,10]. This updated physiological framework has important implications for patient selection, biochemical monitoring, and therapeutic management.

After reinfusion into the systemic circulation, citrate is rapidly metabolized through mitochondrial oxidative pathways, predominantly in the liver but also in skeletal muscle and the renal cortex [5,10,11]. Oxidation of citrate within the tricarboxylic acid (TCA) cycle generates bicarbonate equivalents while releasing calcium from calcium–citrate complexes. Consequently, citrate metabolism depends primarily on preserved mitochondrial oxidative capacity across several organs rather than on hepatic function alone [7,8,9,10,12]. This concept has progressively replaced the traditional view that liver dysfunction is the principal determinant of citrate intolerance and now forms the physiological basis for contemporary RCA risk stratification.

Systemic citrate accumulation develops when citrate delivery exceeds the combined capacity for metabolic degradation and extracorporeal removal. Conditions associated with impaired oxidative metabolism—notably circulatory shock, severe tissue hypoperfusion, persistent hyperlactatemia, hypothermia, or excessive citrate delivery—reduce systemic citrate clearance and increase the likelihood of accumulation [10,11,13]. In contrast, severe liver dysfunction alone is often well tolerated when adequate tissue perfusion and mitochondrial function are preserved [5,8,9]. Safe RCA also requires a clear distinction between systemic and post-filter ionized calcium monitoring. While systemic ionized calcium reflects the patient’s calcium homeostasis, post-filter ionized calcium primarily reflects the intensity of regional anticoagulation and should be maintained within the recommended therapeutic range (approximately 0.20–0.40 mmol/L). Values substantially below this range increase citrate delivery without necessarily improving circuit anticoagulation, thereby potentially increasing systemic citrate exposure.

Citrate accumulation produces a characteristic biochemical profile that typically precedes overt clinical manifestations. Progressive ionized hypocalcemia, an increasing total-to-ionized calcium (T/iCa) ratio, escalating calcium infusion requirements, high anion-gap metabolic acidosis, and frequently hyperlactatemia provide early evidence of impaired citrate handling and should prompt timely therapeutic intervention before clinical deterioration occurs [10,13,14,15].

The reported incidence of citrate accumulation varies considerably according to patient population, RCA protocol, and the definition applied, ranging from approximately 5% to 25% during RCA-CKRT, with the highest incidence observed in patients with circulatory shock or multiple organ failure [10,11]. Throughout this review, clinically relevant citrate accumulation refers to persistent systemic citrate retention associated with a T/iCa ratio > 2.5 together with ionized hypocalcemia and high anion-gap metabolic acidosis, irrespective of the presence or absence of overt clinical manifestations [14,15,16].

The aim of this narrative review is to summarize current evidence regarding the physiology of citrate metabolism, the mechanisms and risk factors of systemic citrate accumulation, its biochemical and clinical manifestations, monitoring strategies, therapeutic management, current controversies, and future research directions, with particular emphasis on the physiological concept that citrate intolerance reflects impaired systemic oxidative metabolism rather than isolated hepatic dysfunction.

## 2. Methods

This narrative review was conducted to summarize the current evidence regarding the pathophysiology, diagnosis, monitoring, prevention, and management of systemic citrate accumulation during regional citrate anticoagulation (RCA) for continuous kidney replacement therapy (CKRT). A comprehensive literature search was performed using PubMed/MEDLINE, Embase, and Google Scholar. The search strategy included combinations of the following keywords: “regional citrate anticoagulation”, “continuous kidney replacement therapy”, “continuous renal replacement therapy”, “citrate accumulation”, “citrate toxicity”, “hypocalcemia”, “calcium ratio”, “mitochondrial dysfunction”, “liver dysfunction”, and “shock”. Reference lists of relevant articles and international guidelines were also screened to identify additional publications. Particular emphasis was placed on randomized controlled trials, prospective observational studies, international guidelines, expert consensus statements, and major narrative reviews published in English. Older landmark studies were included when considered essential for understanding the evolution of current concepts regarding citrate metabolism and systemic citrate accumulation. Because this is a narrative review, no formal systematic review methodology or quantitative meta-analysis was performed.

A generative artificial intelligence tool (ChatGPT, GPT-5.6 Sol, OpenAI, San Francisco, CA, USA) was used during manuscript preparation to assist with language editing, structural organization, and the development of conceptual figure drafts. All outputs were independently reviewed and verified by the authors against the original scientific literature. Reference selection, interpretation of the evidence, and all final scientific conclusions remained under the sole responsibility of the authors.

## 3. Pathophysiology and Clinical Features

### Mechanistic Basis of Calcium Chelation

Citrate produces regional anticoagulation by reversibly chelating ionized calcium within the extracorporeal circuit, thereby inhibiting calcium-dependent activation of the coagulation cascade while largely preserving systemic coagulation. Because calcium–citrate complexes subsequently return to the patient, systemic calcium homeostasis depends on the balance between citrate metabolism and extracorporeal citrate removal rather than on citrate infusion alone [9,17].

Following systemic reinfusion, citrate is metabolized through mitochondrial oxidative pathways predominantly in the liver, but also in skeletal muscle and the renal cortex [10,13,14]. Within the tricarboxylic acid (TCA) cycle, citrate oxidation generates bicarbonate while releasing previously chelated calcium, thereby contributing to both calcium homeostasis and acid–base balance [10,13,14]. Experimental and clinical evidence demonstrates that extrahepatic tissues contribute substantially to citrate metabolism, explaining why isolated hepatic dysfunction rarely results in clinically significant citrate accumulation when global oxidative metabolism and tissue perfusion remain preserved [3,5,8,10,13,14,18].

When citrate delivery exceeds the combined metabolic and extracorporeal capacity for citrate elimination, unmetabolized citrate progressively accumulates in the systemic circulation [4,9,10,19]. Persistent circulating citrate continues to chelate ionized calcium and, to a lesser extent, magnesium, resulting in progressive ionized hypocalcemia despite stable or even increased total serum calcium concentrations [9,10,16,20]. This dissociation between total and ionized calcium is reflected by an increasing total-to-ionized calcium (T/iCa) ratio, which remains the most widely accepted surrogate marker of systemic citrate accumulation [10,20,21].

Post-filter ionized calcium primarily reflects the intensity of regional anticoagulation rather than systemic citrate metabolism. Achieving lower post-filter ionized calcium concentrations requires greater citrate delivery to the extracorporeal circuit, thereby increasing the systemic citrate load returning to the patient. Consequently, targeting post-filter ionized calcium values substantially below the recommended therapeutic range (0.20–0.40 mmol/L) may unnecessarily increase citrate exposure without providing additional anticoagulant benefit. Post-filter ionized calcium should therefore be regarded not only as a marker of circuit anticoagulation but also as an important determinant of systemic citrate delivery [7,8,22,23,24].

In parallel, retained citrate behaves as a strong unmeasured anion, promoting high anion-gap metabolic acidosis when citrate delivery exceeds the patient’s metabolic capacity and extracorporeal citrate clearance [4,9,10,19]. Together, ionized hypocalcemia, an increasing T/iCa ratio, escalating calcium infusion requirements, and metabolic acidosis constitute the characteristic biochemical signature of systemic citrate accumulation and provide the physiological basis for its diagnosis, monitoring, and management [4,10,14,19].

**Figure 1:**
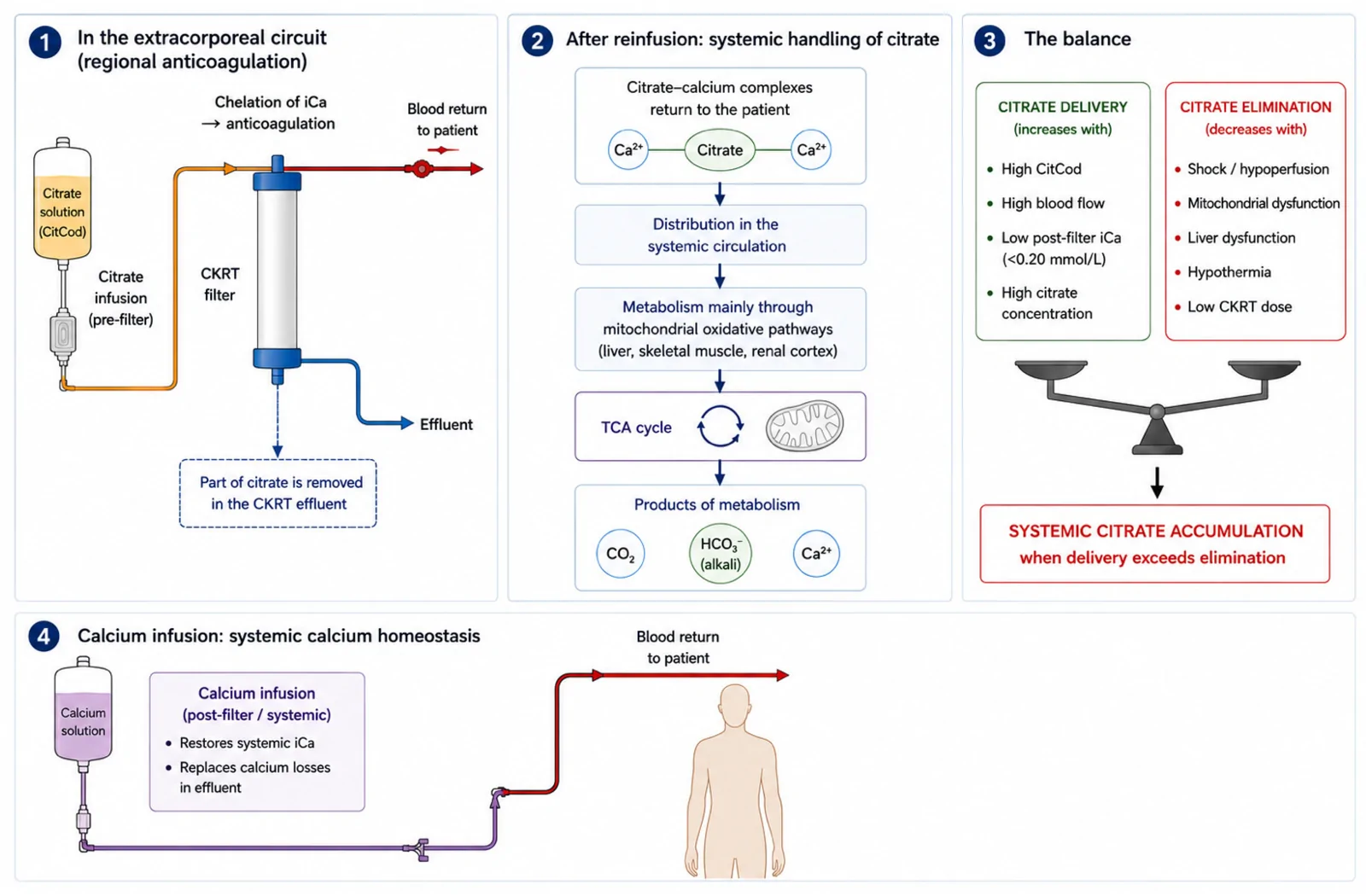
Physiological basis of regional citrate anticoagulation and systemic citrate accumulation during continuous kidney replacement therapy. Legend: Overview of citrate delivery, metabolism, and elimination during regional citrate anticoagulation. Citrate chelates ionized calcium within the extracorporeal circuit to provide anticoagulation, while calcium infusion maintains systemic calcium homeostasis. Systemic citrate accumulation develops when citrate delivery exceeds the patient’s metabolic and extracorporeal capacity for citrate elimination. Abbreviations: ATP, adenosine triphosphate; CitCod, citrate dose; CKRT, continuous kidney replacement therapy; CO_2_, carbon dioxide; HCO_3_^−^, bicarbonate; iCa, ionized calcium; Mg^2+^, ionized magnesium; RCA, regional citrate anticoagulation; TCA, tricarboxylic acid; T/iCa, total-to-ionized calcium ratio. GenAI statement: This conceptual figure was developed with the assistance. ChatGPT (OpenAI, San Francisco, CA, USA; https://chatgpt.com/; accessed on 20 June 2026) and subsequently reviewed, revised, and validated by the authors.

Collectively, these mechanisms demonstrate that systemic citrate accumulation reflects a dynamic imbalance between citrate delivery and the patient’s combined metabolic and extracorporeal capacity for citrate elimination rather than isolated hepatic dysfunction. This physiological framework underpins the risk factors, biochemical abnormalities, monitoring strategies, and therapeutic interventions discussed throughout this review. The principal determinants of citrate delivery, metabolism, and elimination are summarized in Figure 1.

## 4. Risk Factors for Citrate Accumulation

### 4.1. Major Risk Factors

Circulatory shock is the strongest and most consistent predictor of systemic citrate accumulation during regional citrate anticoagulation (RCA) [10,11,13]. By impairing tissue oxygen delivery, shock reduces mitochondrial oxidative phosphorylation and tricarboxylic acid (TCA) cycle activity across multiple organs, thereby decreasing the patient’s overall capacity to metabolize citrate [10,13]. Consequently, citrate delivery may exceed the combined metabolic and extracorporeal capacity for citrate elimination, resulting in progressive systemic citrate accumulation [10,13]. This phenomenon reflects global impairment of oxidative metabolism rather than reduced hepatic perfusion alone, explaining why circulatory shock has emerged as the principal physiological determinant of citrate intolerance [10,13]. The coexistence of shock and severe liver dysfunction further reduces metabolic reserve and markedly increases the risk of citrate accumulation [3,10,11,13,14,18].

Liver dysfunction should therefore no longer be regarded as an absolute contraindication to RCA [3,5,8]. Instead, the risk of citrate accumulation depends on the interaction between hepatic function, circulatory status, and the patient’s global metabolic reserve [10,13]. Clinical and pharmacokinetic studies have shown that many patients with stable liver disease maintain adequate citrate clearance, whereas patients with severe circulatory failure may develop citrate accumulation despite relatively preserved hepatic function [5,8,9,11,25]. These observations support individualized biochemical monitoring rather than excluding patients from RCA solely on the basis of liver dysfunction [26].

Excessive citrate delivery may transiently overwhelm citrate elimination even when mitochondrial oxidative capacity is preserved [10]. High blood flow rates, excessive citrate infusion, or insufficient CKRT effluent doses all increase systemic citrate exposure [10,22]. This condition, commonly referred to as citrate overload, should be distinguished from true systemic citrate accumulation caused by impaired citrate metabolism. In citrate overload, metabolic capacity remains intact, and biochemical abnormalities usually resolve rapidly after reducing citrate delivery or increasing extracorporeal citrate removal [10,22]. Distinguishing these two conditions is clinically important because their pathophysiology and management differ substantially.

### 4.2. Moderate Risk Factors

Persistent hyperlactatemia reflects impaired tissue perfusion and reduced mitochondrial oxidative capacity rather than citrate accumulation itself [10,13]. Although elevated lactate frequently accompanies citrate accumulation, it lacks sufficient sensitivity and specificity to establish the diagnosis [10]. Instead, lactate should be interpreted as a marker of reduced metabolic reserve that identifies patients at increased risk of impaired citrate metabolism [10,13,19,24,27].

Hypothermia decreases the activity of several mitochondrial enzymes involved in the TCA cycle, thereby slowing citrate oxidation [28]. Although hypothermia alone rarely causes clinically significant citrate accumulation, it may further reduce metabolic capacity in critically ill patients with circulatory shock or multiple organ dysfunction, thereby increasing the risk of citrate intolerance [10,28].

Reduced CKRT effluent flow limits extracorporeal citrate removal and contributes to systemic citrate exposure when metabolic clearance is already compromised [10,22]. While rarely sufficient to induce citrate accumulation in isolation, inadequate effluent flow may aggravate citrate retention in patients with impaired oxidative metabolism and should therefore be considered when adjusting RCA prescriptions [10,22] (Table 1).

## 5. Biochemical Profile of Citrate Accumulation

Systemic citrate accumulation produces a characteristic constellation of biochemical abnormalities that generally precedes overt clinical manifestations. These changes result from impaired citrate metabolism, persistent formation of calcium–citrate and magnesium–citrate complexes, and the accumulation of citrate as a strong unmeasured anion [9,10,20,21]. Consequently, diagnosis should rely on the integrated interpretation of complementary biochemical parameters rather than on any single laboratory measurement considered in isolation [4,8,10,19].

### 5.1. Systemic Ionized Hypocalcemia

Systemic ionized hypocalcemia is the earliest and most sensitive biochemical manifestation of systemic citrate accumulation. Rather than reflecting true calcium depletion, it results from persistent chelation of circulating ionized calcium by unmetabolized citrate when citrate delivery exceeds the patient’s combined metabolic and extracorporeal capacity for citrate elimination [9,10,20,21].

As the burden of circulating calcium–citrate complexes increases, the biologically active ionized calcium fraction progressively declines, whereas total serum calcium remains stable or may even increase because calcium remains bound to citrate [9,10,20]. This physiological dissociation forms the basis of the progressive increase in the total-to-ionized calcium (T/iCa) ratio, the most widely accepted surrogate marker of clinically significant citrate accumulation [10,20,21].

Progressive ionized hypocalcemia may impair myocardial contractility, reduce vascular tone, and increase neuromuscular excitability, potentially leading to cardiac arrhythmias, muscle cramps, tetany, or seizures [10,29]. However, these clinical manifestations usually develop only after substantial biochemical abnormalities have already occurred and are frequently masked in critically ill patients receiving sedation, mechanical ventilation, or neuromuscular blockade [10,29].

Accordingly, ionized hypocalcemia should be regarded primarily as an early biochemical warning sign rather than a late clinical manifestation of citrate accumulation. Persistent or worsening hypocalcemia despite appropriate calcium replacement should prompt immediate reassessment of citrate delivery, extracorporeal citrate removal, tissue perfusion, and the patient’s metabolic reserve [4,8,10,19]. Importantly, normalization of systemic ionized calcium following calcium administration does not indicate resolution of citrate accumulation because it corrects hypocalcemia without reducing the underlying citrate burden. Therefore, systemic ionized calcium should always be interpreted together with the T/iCa ratio, calcium infusion requirements, acid–base status, and serial biochemical trends rather than as an isolated laboratory value [4,8,10,19,20].

### 5.2. Total-to-Ionized Calcium Ratio (T/iCa Ratio)

The total-to-ionized calcium (T/iCa) ratio is the most widely accepted surrogate marker of systemic citrate accumulation during regional citrate anticoagulation because it reflects the progressive formation of circulating calcium–citrate complexes as citrate metabolism becomes impaired [10,20,21]. As systemic citrate accumulates, an increasing proportion of total serum calcium remains bound to citrate, resulting in a progressive decline in ionized calcium while total calcium remains stable or may even increase [9,10,20]. Consequently, the T/iCa ratio provides an indirect estimate of impaired citrate metabolism rather than a direct measurement of circulating citrate concentrations [10,20,21].

A T/iCa ratio > 2.5 (when calcium concentrations are expressed in mmol/L) is widely considered suggestive of clinically significant citrate accumulation and remains incorporated into most contemporary RCA monitoring protocols [10,20,21,30]. However, this threshold should be regarded as a practical guide rather than an absolute diagnostic criterion. Its diagnostic performance may be influenced by several factors, including hypoalbuminemia, alterations in total calcium concentration, analytical methodology, and the dynamic nature of citrate metabolism [16,19]. For these reasons, the T/iCa ratio should never be interpreted in isolation.

Current expert recommendations advocate integrating the T/iCa ratio with systemic ionized calcium, calcium infusion requirements, acid–base status, lactate concentrations, and the overall clinical context to improve diagnostic accuracy and reduce unnecessary interruption of RCA [4,7,8,19]. Serial changes in the T/iCa ratio are generally more informative than isolated measurements, particularly during treatment initiation or following adjustments to the RCA prescription [4,10,19]. Conversely, a mildly elevated T/iCa ratio in an otherwise stable patient does not necessarily indicate clinically significant citrate accumulation requiring citrate discontinuation. Clinical interpretation should therefore rely on the overall biochemical trajectory rather than a single threshold value.

#### 5.2.1. Metabolic Acidosis

Metabolic acidosis is one of the hallmark biochemical manifestations of systemic citrate accumulation and reflects impaired citrate metabolism rather than citrate administration itself [10,14,15]. Under physiological conditions, citrate returning to the systemic circulation is rapidly metabolized through the tricarboxylic acid (TCA) cycle, generating bicarbonate that contributes to systemic buffering and may even result in mild metabolic alkalosis during appropriately prescribed RCA [10,14,15].

When citrate delivery exceeds the patient’s combined metabolic and extracorporeal capacity for citrate elimination, unmetabolized citrate accumulates as a strong unmeasured anion, increasing the anion gap and producing high anion-gap metabolic acidosis [9,10,20,21]. The severity of acidosis generally parallels the impairment of citrate metabolism and is usually accompanied by progressive ionized hypocalcemia, an increasing total-to-ionized calcium (T/iCa) ratio, and rising calcium infusion requirements [4,10,20,21]. Together, these abnormalities constitute the characteristic biochemical profile of clinically significant systemic citrate accumulation.

Nevertheless, metabolic acidosis should never be interpreted in isolation. Critically ill patients frequently have multiple concurrent causes of acid–base disturbances, including lactic acidosis, ketoacidosis, renal failure, and hyperchloremic acidosis related to fluid therapy [4,8,10,19]. Consequently, worsening acidosis during RCA should prompt a comprehensive assessment of the patient’s metabolic status rather than immediate attribution to citrate accumulation alone.

Conversely, restoration of tissue perfusion and mitochondrial oxidative capacity re-establishes efficient citrate metabolism, allowing retained citrate to be converted into bicarbonate. This delayed bicarbonate generation may occasionally result in transient rebound metabolic alkalosis following recovery from systemic citrate accumulation or correction of the underlying circulatory disorder [10,14,15]. Accordingly, acid–base disturbances during RCA should be interpreted as dynamic markers of citrate metabolism rather than direct consequences of citrate administration.

#### 5.2.2. Systemic Ionized Hypomagnesemia

Systemic ionized hypomagnesemia is a frequent but often underrecognized biochemical consequence of regional citrate anticoagulation. Similar to calcium, magnesium readily binds citrate to form soluble magnesium–citrate complexes. When citrate metabolism becomes impaired, circulating ionized magnesium progressively decreases, whereas total magnesium concentrations may remain within the normal range because an increasing proportion of magnesium is citrate-bound [9,10,16]. Consequently, ionized magnesium, whenever available, provides a more physiologically relevant assessment of magnesium homeostasis than total magnesium.

Magnesium plays an essential role in myocardial electrophysiology, vascular tone, neuromuscular transmission, and numerous enzymatic reactions. Progressive ionized hypomagnesemia may therefore contribute to cardiac arrhythmias, impaired myocardial contractility, muscle weakness, tremor, and neuromuscular hyperexcitability, particularly in critically ill patients with multiple concurrent electrolyte disturbances [10,16,29]. In addition, magnesium deficiency may impair parathyroid hormone secretion and reduce tissue responsiveness to parathyroid hormone, thereby aggravating hypocalcemia and making restoration of calcium homeostasis more difficult [16].

Despite these physiological effects, ionized hypomagnesemia is not currently considered a primary diagnostic marker of systemic citrate accumulation [4,19]. Instead, it should be interpreted as a complementary biochemical abnormality within the overall metabolic profile. Serial monitoring of magnesium—preferably ionized magnesium when available—is particularly relevant in patients with persistent hypocalcemia, refractory arrhythmias, or unexplained neuromuscular manifestations. Magnesium supplementation may facilitate correction of associated electrolyte disturbances but should always complement, rather than replace, optimization of citrate delivery, extracorporeal citrate removal, and calcium replacement [10,19,29].

#### 5.2.3. Hypernatremia

Hypernatremia was historically recognized as a potential complication of regional citrate anticoagulation because early RCA protocols relied on hypertonic trisodium citrate solutions that delivered substantial sodium loads [9,10,31]. Under these conditions, excessive sodium administration frequently resulted in hypernatremia, particularly when dialysis or replacement fluid prescriptions were insufficient to compensate for the sodium burden.

Contemporary RCA protocols differ substantially from these early strategies. Most commercially available citrate solutions are specifically formulated to minimize sodium loading and are combined with standardized dialysate and replacement fluid prescriptions that maintain overall sodium balance [4,19]. Consequently, clinically significant hypernatremia has become uncommon during modern RCA and, when present, more often reflects disturbances in sodium balance, inadequate free-water administration, or inappropriate CKRT prescription than impaired citrate metabolism itself [4,10,19].

**Figure 2:**
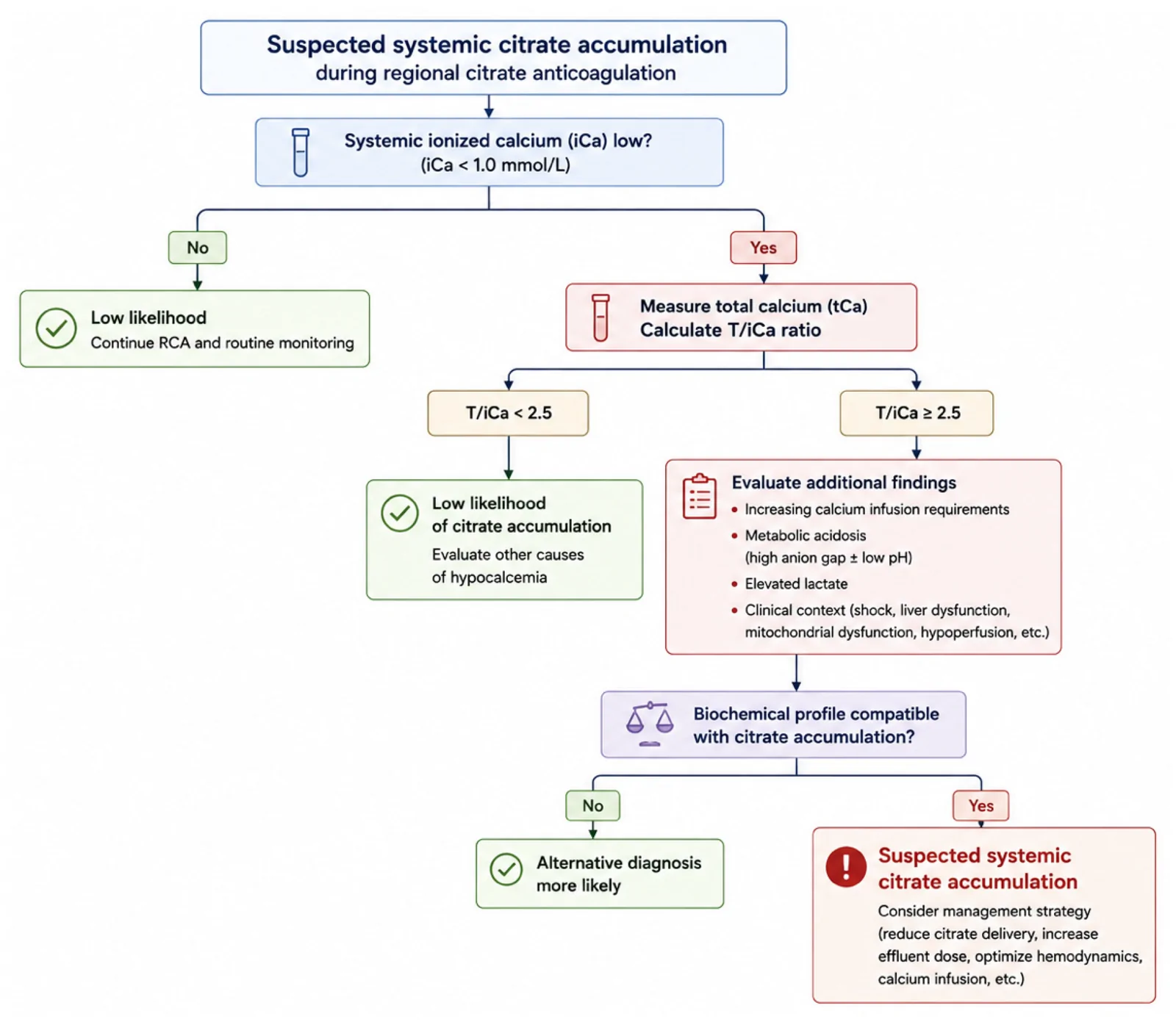
Diagnostic approach to suspected systemic citrate accumulation during regional citrate anticoagulation. Legend: Simplified diagnostic algorithm for suspected systemic citrate accumulation during RCA. Evaluation is based on systemic ionized calcium, the total-to-ionized calcium ratio, complementary biochemical findings, and the clinical context, with alternative diagnoses considered when the overall profile is not compatible with citrate accumulation. Abbreviations: iCa, ionized calcium; RCA, regional citrate anticoagulation; tCa, total calcium; T/iCa, total-to-ionized calcium ratio. GenAI statement: This diagnostic algorithm was developed with the assistance of ChatGPT (OpenAI) and subsequently reviewed, revised, and validated by the authors.

Accordingly, isolated hypernatremia should no longer be regarded as a diagnostic marker of systemic citrate accumulation. Instead, serum sodium should be interpreted within the overall biochemical and clinical context together with systemic ionized calcium, the total-to-ionized calcium (T/iCa) ratio, acid–base status, and calcium infusion requirements [4,10,19]. Routine monitoring of serum sodium nevertheless remains an important component of RCA management because it facilitates early recognition of treatment-related electrolyte disturbances and appropriate adjustment of the extracorporeal prescription [10,19,22] (Table 2; Figure 2).

#### 5.2.4. Clinical Manifestations

Clinical manifestations of systemic citrate accumulation usually occur late in the course of metabolic deterioration and are almost invariably preceded by progressive biochemical abnormalities [4,10,19]. Consequently, diagnosis should rely primarily on structured biochemical monitoring rather than waiting for overt clinical manifestations, which generally indicate advanced impairment of citrate metabolism.

The cardiovascular system is most commonly affected. Progressive ionized hypocalcemia may impair myocardial contractility, prolong ventricular repolarization, and contribute to hypotension, ventricular arrhythmias, and hemodynamic instability [10,29]. QT interval prolongation and both supraventricular and ventricular arrhythmias may occur, particularly in patients with pre-existing cardiac disease or concomitant electrolyte disturbances [10,29]. However, these findings are common in critically ill patients and should therefore be interpreted in conjunction with the characteristic biochemical profile of citrate accumulation rather than as isolated clinical indicators.

Neuromuscular manifestations result from increased neuronal excitability secondary to ionized hypocalcemia. In awake patients, symptoms may include circumoral paresthesia, distal numbness, muscle cramps, carpopedal spasm, tetany, and, in severe cases, generalized seizures [10,29]. In the intensive care setting, however, these manifestations are frequently masked by sedation, mechanical ventilation, or neuromuscular blockade, limiting their diagnostic value.

Persistent high anion-gap metabolic acidosis may further aggravate hemodynamic instability but reflects impaired citrate metabolism rather than a direct toxic effect of citrate [10,14,15]. No individual clinical manifestation is specific for systemic citrate accumulation. Instead, the diagnosis should be suspected when otherwise unexplained cardiovascular deterioration or refractory electrolyte abnormalities occur together with progressive ionized hypocalcemia, an increasing total-to-ionized calcium (T/iCa) ratio, rising calcium infusion requirements, and worsening metabolic acidosis [4,10,19]. Because biochemical abnormalities consistently precede clinical manifestations, early recognition depends on integrated interpretation of serial biochemical trends rather than clinical findings alone (Table 3).

#### 5.2.5. Monitoring Strategies

Early recognition of systemic citrate accumulation relies primarily on structured biochemical monitoring because clinical manifestations are often delayed, nonspecific, or masked in critically ill patients [4,8,10,19]. No single laboratory parameter is sufficiently sensitive or specific to establish the diagnosis. Instead, monitoring should integrate serial biochemical measurements with the patient’s hemodynamic status, tissue perfusion, metabolic reserve, and overall clinical evolution.

Systemic ionized calcium remains the cornerstone of routine monitoring during RCA because it is usually the earliest biochemical parameter to change when citrate metabolism becomes impaired [4,10,19,20]. However, isolated ionized calcium measurements should not be interpreted alone. Progressive hypocalcemia may result from several conditions encountered in critically ill patients, including sepsis, respiratory alkalosis, massive transfusion, and other electrolyte disorders. Conversely, normalization of ionized calcium following calcium infusion does not necessarily indicate recovery of citrate metabolism because calcium replacement corrects hypocalcemia without reducing the underlying citrate burden [4,10,19,20].

The total-to-ionized calcium (T/iCa) ratio remains the most widely accepted surrogate marker of systemic citrate accumulation because it reflects the progressive formation of circulating calcium–citrate complexes [10,20,21]. Nevertheless, its interpretation requires clinical context. Early citrate accumulation may occur before a significant increase in the T/iCa ratio, whereas mildly elevated ratios do not necessarily indicate clinically relevant toxicity [16,19]. Accordingly, the T/iCa ratio should always be interpreted together with systemic ionized calcium, acid–base status, calcium infusion requirements, and serial biochemical trends [4,8,10,19].

Acid–base status provides valuable complementary information because progressive high anion-gap metabolic acidosis reflects impaired citrate metabolism once alternative causes have been reasonably excluded [4,10,19]. Likewise, increasing calcium infusion requirements despite persistent hypocalcemia represent an early warning sign that citrate delivery may be exceeding the patient’s metabolic and extracorporeal capacity for citrate elimination [4,10,19].

Serum lactate should be interpreted as a marker of impaired tissue perfusion and reduced metabolic reserve rather than as a direct marker of citrate accumulation [10,13,19]. Similarly, ionized magnesium, serum sodium, and other routine electrolyte measurements provide useful complementary information but should not be considered primary diagnostic markers [4,10,19].

Monitoring frequency should be individualized according to the patient’s clinical condition and the phase of RCA. More intensive surveillance is recommended during treatment initiation, after changes in citrate or calcium infusion rates, and in patients with circulatory shock, severe liver dysfunction, or rapidly evolving metabolic abnormalities [4,7,8,19,23,24]. Once biochemical stability has been achieved, monitoring intervals may be progressively extended according to local protocols and serial laboratory trends.

Overall, effective monitoring depends on recognizing dynamic biochemical patterns rather than isolated laboratory abnormalities. Early identification of evolving citrate accumulation through integrated interpretation of serial trends allows timely therapeutic intervention before overt clinical deterioration develops.

A practical summary of the principal biochemical parameters and their recommended interpretation during RCA is presented in Table 4.

#### 5.2.6. Management of Citrate Accumulation

Management of systemic citrate accumulation should follow a stepwise physiological approach aimed at restoring the balance between citrate delivery and its metabolic and extracorporeal elimination. Because citrate accumulation is usually reversible, treatment should initially focus on correcting reversible physiological disturbances before considering discontinuation of regional citrate anticoagulation (RCA). Therapeutic decisions should be guided by serial biochemical trends and the overall clinical context rather than isolated laboratory abnormalities [4,7,8,10,19,24].

1. **General Principles**

Progressively increasing calcium infusion requirements despite stable RCA settings represent one of the earliest practical indicators of evolving citrate accumulation because they reflect persistent calcium chelation by circulating citrate [4,19]. However, calcium requirements should always be interpreted together with systemic ionized calcium, the total-to-ionized calcium (T/iCa) ratio, acid–base status, lactate concentrations, and serial biochemical trends. Since calcium requirements may also vary according to CKRT prescription or patient-related factors, management should rely on the overall biochemical profile rather than on a single parameter [4,10,19].

2. **Identify and Correct the Underlying Cause**

The first therapeutic priority is to identify and correct the physiological disturbance responsible for impaired citrate metabolism. In most patients, citrate accumulation reflects reversible impairment of mitochondrial oxidative metabolism caused by circulatory shock, inadequate tissue perfusion, or severe hypoxemia rather than isolated liver dysfunction [4,7,8,10,19]. Restoration of tissue perfusion, optimization of oxygen delivery, and treatment of the underlying critical illness frequently improve citrate metabolism and allow resolution of citrate accumulation without discontinuing RCA [4,8,10,19]. Although severe liver dysfunction may contribute to impaired citrate metabolism, it should generally be regarded as an aggravating factor rather than the primary therapeutic target.

3. **Optimize Hemodynamics**

Optimization of hemodynamic status is fundamental because adequate tissue perfusion is essential for mitochondrial oxidative metabolism [10,13]. Management should be individualized and may include fluid resuscitation, optimization of cardiac output, vasopressor or inotropic support, correction of severe anemia or hypoxemia, and treatment of the underlying cause of shock [10,13]. As tissue perfusion improves, citrate metabolism is usually restored, resulting in progressive normalization of ionized calcium, decreasing calcium infusion requirements, improvement of the T/iCa ratio, and correction of metabolic acidosis. These biochemical improvements often precede complete clinical recovery and may allow continuation of RCA [10,13,22].

4. **Reduce Citrate Delivery**

If biochemical abnormalities continue to worsen despite optimization of the patient’s metabolic condition, citrate delivery should be reduced. Depending on the RCA protocol, this may be achieved by decreasing blood flow, lowering the citrate infusion rate, or adjusting CKRT settings while maintaining adequate circuit anticoagulation [4,10,19]. Serial biochemical reassessment is essential after any modification to confirm restoration of calcium homeostasis and improvement of the overall metabolic profile.

5. **Optimize Extracorporeal Citrate Elimination**

Increasing dialysate and/or replacement fluid flow enhances extracorporeal citrate removal and reduces systemic citrate exposure without necessarily interrupting RCA [4,19]. This strategy is particularly useful in patients with partially preserved metabolic capacity, in whom citrate delivery only modestly exceeds citrate elimination. Further adjustments to the CKRT prescription should be guided by serial assessment of systemic ionized calcium, the T/iCa ratio, calcium infusion requirements, and acid–base status [4,7,8,19].

6. **Calcium Replacement**

Systemic calcium replacement is an essential supportive component of RCA but should not be considered definitive treatment for citrate accumulation. Calcium administration restores circulating ionized calcium concentrations and prevents the consequences of hypocalcemia without reducing the underlying citrate burden [4,7,8,10,19,20]. Accordingly, escalating calcium infusion requirements despite appropriate replacement should prompt reassessment of citrate metabolism and the RCA prescription rather than progressive calcium administration alone [4,7,8,10,19].

7. **Correct Electrolyte and Acid–Base Disturbances**

Correction of electrolyte and acid–base abnormalities should accompany, but never replace, interventions aimed at restoring citrate metabolism [9,10]. Magnesium supplementation should be guided by ionized magnesium concentrations whenever available, particularly in patients with persistent hypocalcemia or refractory arrhythmias [9,16,29]. Severe metabolic acidosis may require bicarbonate therapy; however, bicarbonate corrects acidemia without improving citrate metabolism and should never delay treatment of the underlying physiological disturbance [9,10,22]. Hypernatremia has become uncommon with contemporary RCA protocols and should be managed by optimizing sodium balance and the CKRT prescription rather than by assuming citrate accumulation alone [9,22].

8. **Discontinue Citrate Anticoagulation**

RCA should be discontinued when citrate accumulation persists or progresses despite optimization of tissue perfusion, reduction in citrate delivery, enhancement of extracorporeal citrate elimination, and appropriate supportive therapy [7,8,9,10,19,22]. The decision should always rely on the overall biochemical and clinical picture rather than on any single laboratory abnormality.

Findings supporting discontinuation of RCA include:

- Persistent T/iCa ratio > 3.0 despite optimization of citrate delivery and extracorporeal citrate elimination.
- Persistent systemic ionized hypocalcemia (e.g., <0.7 mmol/L) despite appropriate calcium replacement.
- Progressive high anion-gap metabolic acidosis (e.g., pH < 7.20) despite correction of reversible causes.
- Progressive hemodynamic instability or worsening organ dysfunction attributable to citrate accumulation.
- Persistent deterioration of the overall biochemical profile despite appropriate corrective interventions [9,10,22].

Following discontinuation of RCA, anticoagulation should be individualized according to bleeding risk and local practice. Options include systemic unfractionated heparin, CKRT without anticoagulation in patients at very high bleeding risk, or alternative institutional protocols [7,8,22,32]. Serial biochemical monitoring should continue until normalization of ionized calcium, the T/iCa ratio, calcium infusion requirements, and acid–base status confirms recovery of citrate metabolism. Whenever feasible, correction of reversible physiological disturbances should be prioritized to preserve the well-established benefits of RCA.

**Figure 3:**
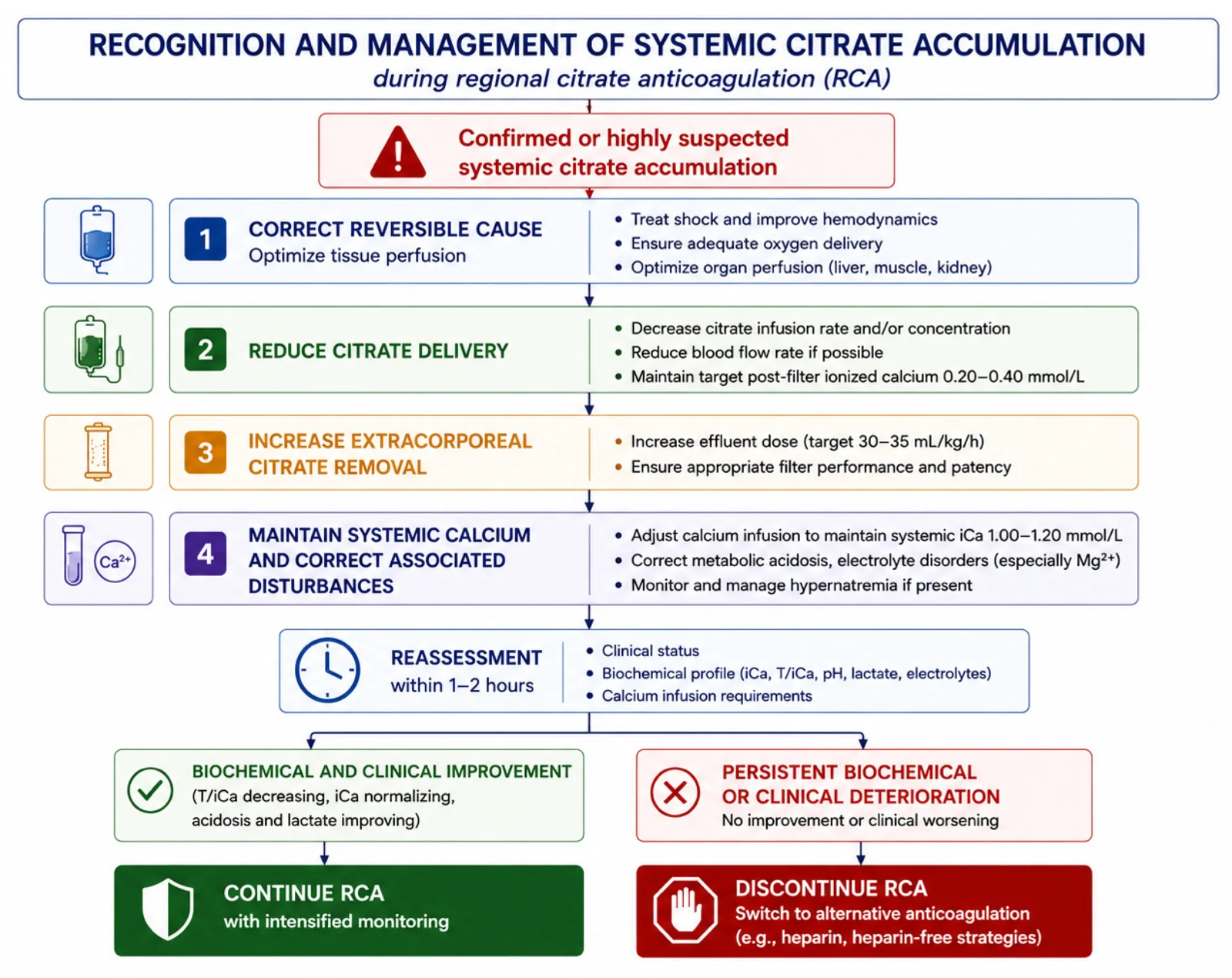
Recognition and management of systemic citrate accumulation during regional citrate anticoagulation. Legend: Stepwise management algorithm for patients with confirmed or highly suspected systemic citrate accumulation during regional citrate anticoagulation. Management focuses on correcting reversible physiological disturbances, restoring the balance between citrate delivery and elimination, and reassessing the biochemical and clinical response before deciding whether RCA can be safely continued or should be discontinued. Abbreviations: iCa, ionized calcium; Mg^2+^, ionized magnesium; RCA, regional citrate anticoagulation; T/iCa, total-to-ionized calcium ratio. GenAI statement: This management algorithm was developed with the assistance of ChatGPT (OpenAI) and subsequently reviewed, revised, and validated by the authors.

A practical algorithm summarizing the recognition, monitoring, and management of systemic citrate accumulation is presented in Figure 3.

## 6. Discussion

Although systemic citrate accumulation remains the principal metabolic complication of regional citrate anticoagulation (RCA), contemporary evidence indicates that it has become considerably less frequent with the implementation of standardized RCA protocols and structured biochemical monitoring [2,3,4,8,10]. One of the most important advances in recent years has been the recognition that citrate accumulation should no longer be viewed as an isolated consequence of liver dysfunction. Instead, it reflects a dynamic physiological imbalance in which citrate delivery exceeds the patient’s combined metabolic and extracorporeal capacity for citrate elimination [3,4,7,8,10]. This concept provides a coherent physiological framework for understanding risk factors, interpreting biochemical abnormalities, and guiding therapeutic management during RCA [14,15,18,19].

This evolving understanding has important implications for clinical practice. Experimental, physiological, and clinical studies consistently demonstrate that citrate metabolism is distributed across several organs, with substantial contributions from skeletal muscle and the renal cortex in addition to the liver [3,5,8,10,14,18]. Consequently, many patients with stable liver disease retain sufficient citrate-metabolizing capacity to tolerate RCA without clinically significant citrate accumulation [5,8,9]. In contrast, circulatory shock has emerged as the principal determinant of impaired citrate metabolism because inadequate tissue oxygen delivery compromises mitochondrial oxidative function simultaneously across multiple organs, thereby reducing global metabolic capacity [10,13]. Risk assessment should therefore extend beyond static evaluation of liver function and incorporate hemodynamic status, tissue perfusion, and markers of metabolic reserve, which together provide a more physiologically relevant estimate of the patient’s ability to metabolize citrate [4,8,10,13,19].

The clinically relevant question is therefore no longer whether the liver can metabolize citrate, but whether the patient retains sufficient systemic oxidative capacity to metabolize the citrate load delivered by the extracorporeal circuit. This concept also highlights that excessive lowering of post-filter ionized calcium may unnecessarily increase systemic citrate exposure without improving anticoagulation efficacy, reinforcing the importance of maintaining post-filter ionized calcium within the recommended therapeutic range rather than pursuing unnecessarily low targets.

**Figure 4:**
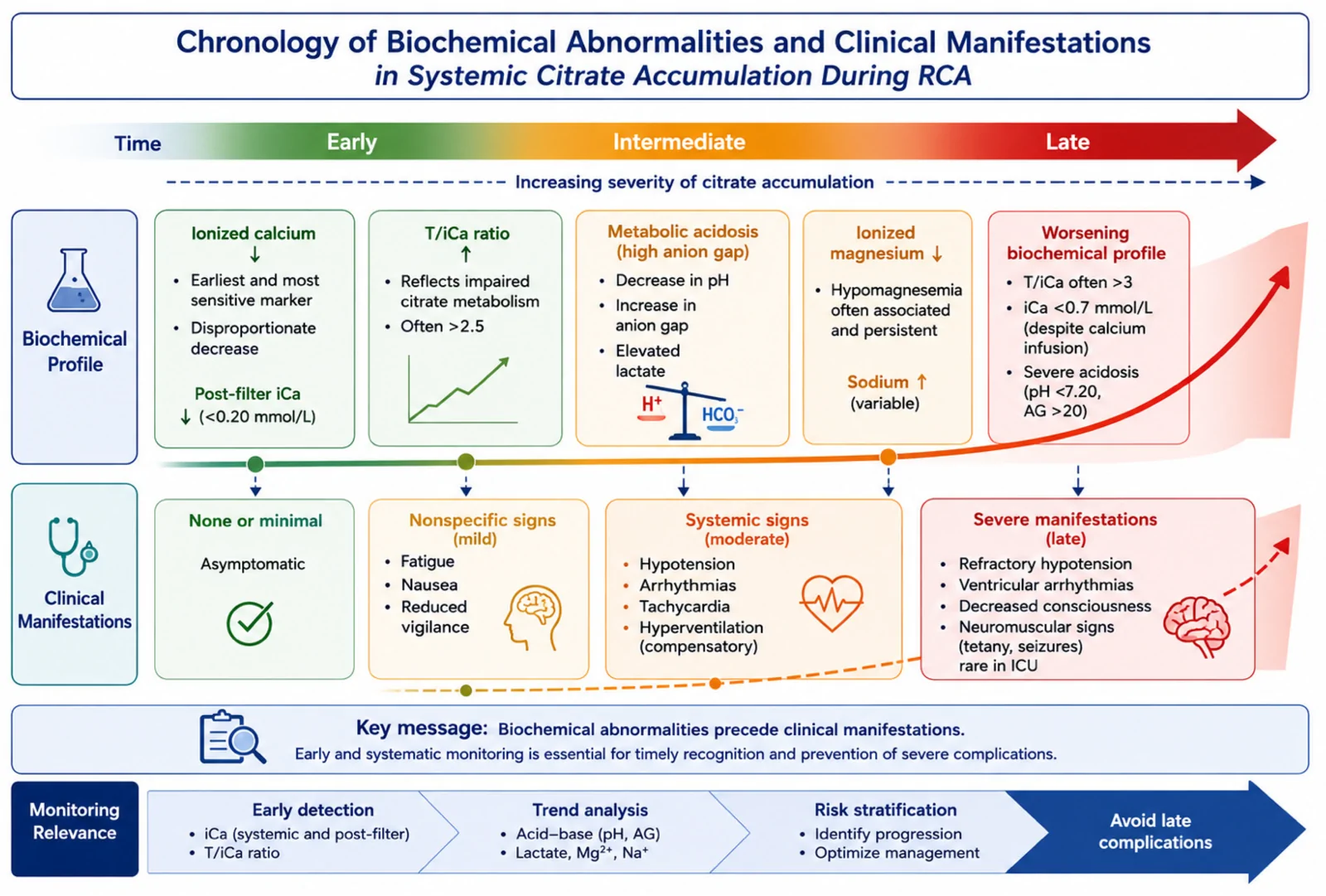
Chronology of biochemical abnormalities and clinical manifestations during systemic citrate accumulation. Legend: Conceptual timeline illustrating the typical progression of systemic citrate accumulation during regional citrate anticoagulation. Biochemical abnormalities generally precede clinical manifestations, emphasizing the importance of serial biochemical monitoring for early recognition and timely intervention. Abbreviations: AG, anion gap; HCO_3_^−^, bicarbonate; iCa, ionized calcium; Mg^2+^, ionized magnesium; Na^+^, sodium; RCA, regional citrate anticoagulation; T/iCa, total-to-ionized calcium ratio. GenAI statement: This conceptual figure was developed with the assistance of ChatGPT (OpenAI) and subsequently reviewed, revised, and validated by the authors.

These physiological concepts also influence the diagnosis and management of systemic citrate accumulation. Current evidence supports an integrated approach based on serial biochemical trends rather than isolated laboratory abnormalities [4,8,10,19]. Although the total-to-ionized calcium (T/iCa) ratio remains the most widely accepted surrogate marker, its diagnostic performance is substantially improved when interpreted together with systemic ionized calcium, calcium infusion requirements, acid–base status, lactate concentrations, and, most importantly, their evolution over time [10,19,20,21]. As illustrated in Figure 4, biochemical abnormalities consistently precede overt clinical manifestations, providing a valuable opportunity for early recognition and intervention. Consequently, structured biochemical monitoring remains the cornerstone of safe RCA practice, particularly in patients with circulatory shock or reduced metabolic reserve [4,8,10,19]. From a therapeutic perspective, these concepts support a physiology-based management strategy focused on restoring the balance between citrate delivery and citrate elimination through optimization of tissue perfusion, individualized adjustment of citrate delivery, enhancement of extracorporeal citrate clearance, and correction of associated metabolic abnormalities. Such an approach allows continuation of RCA in many patients who would previously have undergone unnecessary discontinuation of citrate anticoagulation [2,4,7,8,9,10,19,22].

### 6.1. Strengths and Limitations

This review has several important strengths. First, it integrates contemporary mechanistic, physiological, biochemical, and clinical evidence into a coherent conceptual framework in which systemic citrate accumulation is viewed as a dynamic imbalance between citrate delivery and the patient’s combined metabolic and extracorporeal capacity for citrate elimination rather than as an isolated consequence of hepatic dysfunction [3,4,7,8,9,10]. By synthesizing experimental studies, clinical observations, international guidelines, and expert recommendations, this review bridges current pathophysiological knowledge with practical bedside approaches to risk stratification, diagnosis, monitoring, and management [4,7,8,19]. In addition, the conceptual figures, diagnostic algorithm, and stepwise management strategy provide clinicians with practical tools for translating current evidence into routine RCA practice while emphasizing the integrated interpretation of serial biochemical trends rather than reliance on individual laboratory parameters.

Several limitations should nevertheless be acknowledged. As a narrative rather than a systematic review, this manuscript does not provide a formal quantitative synthesis of the available evidence and cannot completely exclude study selection bias. Furthermore, considerable heterogeneity persists among published studies with respect to RCA protocols, CKRT modalities, citrate formulations, monitoring strategies, and diagnostic definitions of systemic citrate accumulation, limiting direct comparisons and preventing firm conclusions regarding optimal diagnostic thresholds or therapeutic algorithms. Finally, much of the available evidence is derived from physiological studies, single-center observational cohorts, and expert recommendations, whereas prospective multicenter studies specifically designed to validate diagnostic criteria, monitoring strategies, and individualized management strategies remain scarce. Consequently, several concepts proposed in this review—including the integrated physiological framework and the physiology-based management strategy—should be considered evidence-informed interpretations of the current literature rather than definitive standards of care. Their clinical applicability will require confirmation in well-designed prospective multicenter studies.

### 6.2. Current Controversies

Despite substantial advances in the understanding of citrate metabolism, several clinically relevant questions remain unresolved. These controversies reflect the limited availability of prospective physiological studies, the heterogeneity of RCA protocols, and the absence of universally accepted diagnostic criteria for systemic citrate accumulation. Addressing these uncertainties will be essential to further refine individualized RCA strategies and improve patient safety.

#### 6.2.1. Controversy 1: What Is the Optimal T/iCa Threshold?

The optimal total-to-ionized calcium (T/iCa) ratio for diagnosing clinically significant citrate accumulation remains one of the most debated issues in RCA practice because it directly influences therapeutic decision-making. Lower thresholds, such as 2.25, may improve sensitivity for early detection but increase the risk of unnecessary interventions or premature discontinuation of RCA. Conversely, the more widely accepted threshold of 2.5 offers greater specificity but may delay recognition of evolving citrate accumulation in some patients [10,19,24,27].

Honoré and Rimmelé emphasized that the T/iCa ratio should not be interpreted in isolation because its diagnostic performance is influenced by total calcium concentration, albumin levels, and other patient-related factors commonly encountered in critically ill patients [19]. Although these limitations are well recognized, no alternative biomarker has consistently demonstrated superior diagnostic accuracy for routine clinical practice.

Future studies should therefore determine whether serial changes in the T/iCa ratio, interpreted together with systemic ionized calcium, calcium infusion requirements, acid–base status, and clinical evolution, provide greater diagnostic accuracy than any single threshold. Such an approach is consistent with the physiological model proposed throughout this review and may reduce both delayed diagnosis and unnecessary interruption of RCA.

#### 6.2.2. Controversy 2: Can Hyperlactatemia Predict Citrate Accumulation?

The role of lactate as a biomarker of systemic citrate accumulation remains controversial. Although hyperlactatemia frequently accompanies impaired citrate metabolism because both processes are linked to mitochondrial oxidative metabolism and tissue oxygen delivery, lactate is neither sensitive nor specific for citrate accumulation and should not be considered a direct diagnostic marker [10,19,21,24,27].

In critically ill patients, elevated lactate more commonly reflects circulatory shock, impaired tissue perfusion, increased glycolytic activity, or reduced hepatic clearance, all of which may occur independently of citrate metabolism. Conversely, clinically significant citrate accumulation may develop in the absence of marked hyperlactatemia, particularly during the early stages of metabolic impairment or in patients with preserved lactate clearance.

Rather than serving as a diagnostic marker, lactate should be interpreted as an indicator of impaired metabolic reserve and tissue hypoperfusion. Its value lies in identifying patients at increased risk of citrate intolerance when interpreted together with the T/iCa ratio, systemic ionized calcium, acid–base status, calcium infusion requirements, and their temporal evolution [4,10,19,21].

#### 6.2.3. Controversy 3: Is RCA Safe in Advanced Liver Disease Without Shock?

The contribution of liver dysfunction to systemic citrate accumulation remains an area of ongoing debate. Historically, severe liver disease was considered an absolute or near-absolute contraindication to RCA because the liver was believed to be the principal site of citrate metabolism. However, experimental and clinical evidence has fundamentally challenged this paradigm by demonstrating substantial extrahepatic citrate metabolism [3,5,8,10,14,15,18,24].

Current data indicate that skeletal muscle and the renal cortex make major contributions to systemic citrate clearance. Consequently, many patients with chronic liver disease or acute liver dysfunction tolerate RCA without clinically significant citrate accumulation provided that tissue perfusion is preserved and biochemical monitoring is rigorous [3,5,8,9,10,13,18]. Conversely, circulatory shock remains the principal determinant of impaired citrate metabolism because mitochondrial oxidative function becomes compromised simultaneously across multiple organs [10,13,19].

These observations indicate that liver dysfunction should no longer be considered in isolation when evaluating the safety of RCA. Instead, future studies should stratify patients according to both hepatic dysfunction and hemodynamic status rather than considering liver disease as a homogeneous entity. Clinical decision-making should similarly integrate hepatic function, tissue perfusion, mitochondrial oxidative capacity, and extracorporeal citrate elimination when estimating the patient’s overall ability to metabolize citrate.

#### 6.2.4. Controversy 4: When Should RCA Be Discontinued?

The optimal timing for discontinuing RCA in patients with suspected citrate accumulation remains controversial. Historically, RCA was often discontinued at the first evidence of biochemical abnormalities because citrate accumulation was considered an inevitably progressive and potentially life-threatening complication. However, this approach may unnecessarily deprive patients of the established benefits of RCA, including prolonged filter lifespan and reduced bleeding risk [2,9,10,19].

Current evidence suggests that many episodes of citrate accumulation can be reversed by correcting the underlying physiological disturbance rather than immediately discontinuing citrate anticoagulation. Optimization of tissue perfusion, restoration of hemodynamic stability, individualized reduction in citrate delivery, enhancement of extracorporeal citrate elimination, and correction of associated metabolic abnormalities frequently restore citrate metabolism and allow safe continuation of RCA [4,7,8,10,19,22,24].

Accordingly, RCA should not be discontinued solely on the basis of an isolated biochemical abnormality. Instead, the decision should rely on serial biochemical trends, the patient’s overall clinical condition, and the response to corrective interventions. Persistent deterioration despite appropriate physiological optimization should prompt discontinuation of citrate anticoagulation and implementation of an alternative strategy.

### 6.3. Clinical Implications

The concepts discussed throughout this review have several important implications for clinical practice.

**First**, clinicians should move beyond the traditional liver-centered paradigm when assessing the risk of systemic citrate accumulation. Rather than relying primarily on hepatic dysfunction, risk stratification should incorporate hemodynamic status, tissue perfusion, mitochondrial oxidative capacity, and the contribution of extrahepatic citrate metabolism, all of which determine the patient’s overall ability to metabolize citrate [3,5,8,10,13,18].

**Second**, the diagnosis of systemic citrate accumulation should rely on the integrated interpretation of serial biochemical trends rather than isolated laboratory measurements. Although the total-to-ionized calcium (T/iCa) ratio remains the cornerstone of biochemical assessment, it should always be interpreted together with systemic ionized calcium, calcium infusion requirements, acid–base status, lactate concentrations, and their temporal evolution. Serial trends are generally more informative than isolated measurements [4,8,10,19,20,21].

**Third**, therapeutic management should focus on restoring the physiological balance between citrate delivery and citrate elimination. Whenever possible, clinicians should first correct reversible causes of impaired citrate metabolism by optimizing tissue perfusion and hemodynamic status while adjusting citrate delivery and enhancing extracorporeal citrate removal when appropriate. Early physiological intervention often prevents progression to overt citrate toxicity and avoids unnecessary discontinuation of RCA [4,7,8,10,19,22].

**Finally**, this physiology-based approach supports individualized RCA management that preserves the well-established benefits of citrate anticoagulation while improving patient safety. Applying this integrated framework facilitates earlier recognition of systemic citrate accumulation, supports individualized therapeutic decision-making, reduces unnecessary discontinuation of RCA, and helps maintain effective anticoagulation in patients who can safely continue citrate anticoagulation [2,4,7,8,10,19,22].

### 6.4. Future Research Directions

Although major advances have substantially improved the understanding of systemic citrate accumulation during regional citrate anticoagulation (RCA), several important questions remain unanswered. Future research should move beyond isolated biochemical markers toward individualized physiological assessment that better reflects the dynamic interaction between citrate delivery, metabolic reserve, and extracorporeal citrate elimination.

**First**, prospective multicenter studies are needed to validate standardized diagnostic criteria for systemic citrate accumulation. Future investigations should determine whether integrated interpretation of serial biochemical trends—including systemic ionized calcium, the total-to-ionized calcium (T/iCa) ratio, calcium infusion requirements, acid–base status, and hemodynamic variables—improves diagnostic accuracy compared with reliance on individual laboratory parameters or fixed threshold values [4,8,10,19].

**Second**, additional physiological studies are required to better characterize the determinants of citrate metabolism in critically ill patients. The respective contributions of circulatory shock, mitochondrial dysfunction, liver dysfunction, skeletal muscle metabolism, and extracorporeal citrate clearance should be quantified to improve individualized risk stratification and optimize RCA prescription. Future studies should also stratify patients according to both hepatic dysfunction and hemodynamic status rather than considering liver disease as a homogeneous entity [3,5,8,10,13,18].

**Third**, prospective clinical trials should evaluate individualized physiology-based management strategies. Rather than applying uniform treatment algorithms, therapeutic interventions should be tailored according to the patient’s metabolic reserve, tissue perfusion, and capacity for citrate elimination. Whether this personalized approach improves patient safety, circuit lifespan, and clinically relevant outcomes remains to be determined [4,7,8,19,22].

**Finally**, advances in continuous biochemical monitoring, predictive modeling, metabolomic profiling, and artificial intelligence may facilitate earlier recognition of evolving citrate accumulation through real-time integration of multiple physiological variables. Development and prospective validation of clinical prediction models may ultimately support precision RCA management and further improve the safety and effectiveness of continuous kidney replacement therapy.

**Figure 5:**
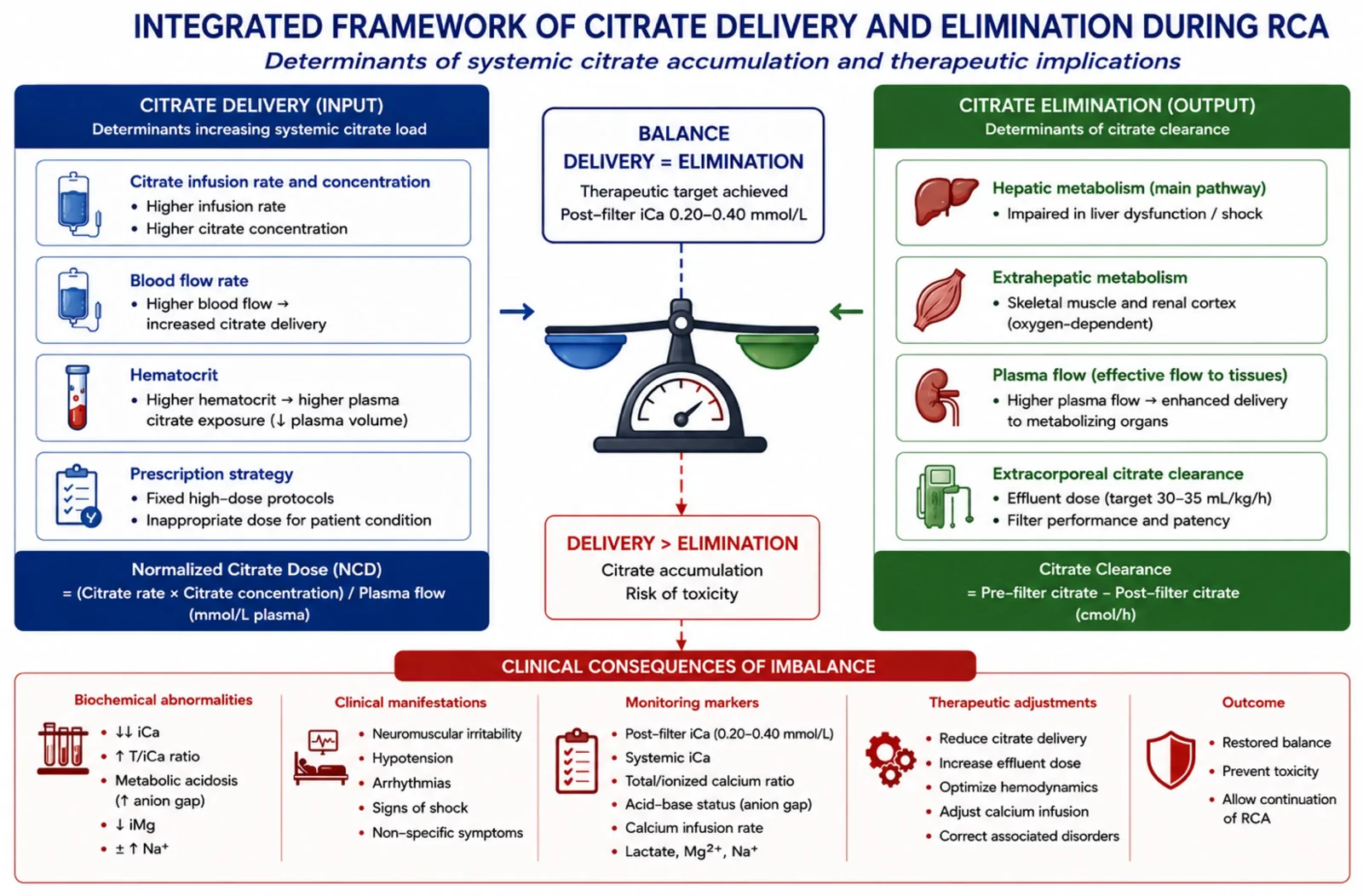
Integrated physiological framework of systemic citrate accumulation during regional citrate anticoagulation. Legend: Conceptual overview illustrating the dynamic balance between citrate delivery and the patient’s combined metabolic and extracorporeal capacity for citrate elimination. Systemic citrate accumulation develops when citrate delivery exceeds citrate elimination, resulting in progressive biochemical abnormalities, subsequent clinical deterioration, and the need for individualized therapeutic interventions aimed at restoring physiological balance. Abbreviations: iCa, ionized calcium; Mg^2+^, ionized magnesium; NCD, normalized citrate dose; Na^+^, sodium; RCA, regional citrate anticoagulation; T/iCa, total-to-ionized calcium ratio. GenAI statement: This conceptual summary figure was developed with the assistance of ChatGPT (OpenAI) and subsequently reviewed, revised, and validated by the authors.

The overall conceptual framework integrating the determinants, pathophysiology, biochemical abnormalities, clinical consequences, monitoring strategies, and therapeutic implications of systemic citrate accumulation is summarized in Figure 5.

## 7. Conclusions

Systemic citrate accumulation remains the principal metabolic complication of regional citrate anticoagulation (RCA) during continuous kidney replacement therapy. Contemporary evidence, however, indicates that it should no longer be viewed primarily as a consequence of hepatic dysfunction but rather as a dynamic imbalance in which citrate delivery exceeds the patient’s metabolic and extracorporeal capacity for citrate elimination. This physiological perspective explains why circulatory shock and impaired tissue perfusion, rather than liver dysfunction alone, are the principal determinants of clinically significant citrate accumulation.

Early recognition depends on structured biochemical monitoring and integrated interpretation of serial biochemical trends. No single laboratory parameter is sufficient for diagnosis; instead, systemic ionized calcium, the total-to-ionized calcium ratio, calcium infusion requirements, acid–base status, and their temporal evolution should be interpreted together to identify evolving citrate accumulation before overt clinical deterioration develops.

Management should therefore focus on restoring the balance between citrate delivery and citrate elimination by correcting reversible physiological disturbances, optimizing tissue perfusion, individualizing citrate delivery, and enhancing extracorporeal citrate removal when appropriate. Discontinuation of RCA should be reserved for patients with persistent or progressive citrate accumulation despite appropriate corrective measures.

Future research should prioritize prospective validation of integrated diagnostic strategies, individualized physiology-based management approaches, and predictive tools capable of identifying evolving citrate accumulation at an earlier stage. Ultimately, citrate intolerance should be regarded as a marker of impaired systemic oxidative metabolism rather than isolated hepatic dysfunction. This evolving physiological framework provides the basis for safer, more individualized RCA practice and has the potential to further improve the effectiveness and safety of continuous kidney replacement therapy.

## Figures and Tables

**Table 1:** Mechanistic factors contributing to citrate accumulation during regional citrate anticoagulation for continuous kidney replacement therapy (RCA-CKRT).

Mechanism/Condition	Primary Physiological Effect	Effect on Citrate Delivery–Elimination Balance	Clinical Consequence
Circulatory shock/impaired tissue perfusion	↓ Oxygen delivery, impaired mitochondrial oxidative phosphorylation	Markedly reduced systemic metabolic capacity for citrate elimination	Rapid systemic citrate accumulation
Persistent hyperlactatemia	Marker of impaired mitochondrial oxidative metabolism and reduced metabolic reserve	Reduced citrate oxidation	Increased risk of citrate accumulation
Severe hepatic dysfunction	Reduced hepatocellular mitochondrial oxidative capacity	Reduced hepatic contribution to citrate metabolism; usually clinically significant only when associated with circulatory failure	Amplifies the effects of shock and reduced metabolic reserve
Hypothermia	Reduced mitochondrial enzyme activity (Q10 effect)	Slowed citrate oxidation	Increased susceptibility to citrate accumulation
Excess citrate delivery/insufficient CKRT effluent	Citrate delivery exceeds extracorporeal elimination	Increased systemic citrate burden despite preserved metabolic capacity	Citrate overload or systemic citrate accumulation, depending on metabolic reserve

Legend. Principal physiological mechanisms that alter citrate delivery, metabolism, or extracorporeal elimination during RCA-CKRT. Abbreviations: CKRT, continuous kidney replacement therapy; RCA, regional citrate anticoagulation. Symbols: ↓, decreased.

**Table 2:** Biochemical signature of systemic citrate accumulation during regional citrate anticoagulation for continuous kidney replacement therapy (RCA-CKRT).

Parameter	Mechanism	Effect/Threshold	Clinical Significance
Ionized Ca^2+^ ↓	Chelation → Ca–citrate complexes	Often <0.9 mmol/L	Early biochemical warning sign; often precedes symptoms
Total Ca ↑	Accumulation of complexed Ca	—	Indicates increasing citrate burden
T/iCa ratio ↑	Ionized Ca decreases while total rises	>2.5 commonly used threshold	Widely used surrogate; interpret with serial biochemical profile
Mg^2+^ ↓ (ionized)	Citrate binds magnesium	—	Promotes arrhythmias & instability
Metabolic acidosis (AG ↑)	Unmetabolized citrate = strong anion	HCO_3_^−^ ↓, AG ↑	Reflects impaired citrate metabolism when alternative causes are excluded
Hypernatremia	Sodium load from citrate solution and/or CKRT prescription imbalance	Na^+^ ↑	Treatment-related electrolyte disturbance; not a specific marker of citrate accumulation
Calcium infusion requirement ↑	Progressive calcium chelation by citrate	Increasing calcium replacement despite unchanged citrate dose	Early warning sign of citrate accumulation

Legend. Principal biochemical abnormalities associated with systemic citrate accumulation; findings should be interpreted as a serial, integrated profile [10,19,20,21,24,27]. Abbreviations: AG, anion gap; CKRT, continuous kidney replacement therapy; HCO_3_^−^, bicarbonate; RCA, regional citrate anticoagulation; T/iCa, total-to-ionized calcium ratio. Symbols: ↑, increased; ↓, decreased; Ca^2+^, calcium; Mg^2+^, magnesium; Na^+^, sodium.

**Table 3:** Integrated interpretation of biochemical and clinical findings suggestive of systemic citrate accumulation during RCA.

Parameter	Isolated Interpretation	Integrated Interpretation
Ionized calcium	Nonspecific alone	Interpret alongside T/iCa, acidosis and calcium requirements
T/iCa ratio	Widely used surrogate	To be interpreted with clinical context
Acidosis	Many possible causes	Becomes suggestive with hypocalcemia and elevated T/iCa
Lactate	Marker of impaired perfusion/metabolic reserve	Supports risk assessment; not a direct marker of citrate accumulation
Clinical signs	Often delayed	Do not wait for their appearance to act

Integrated interpretation of biochemical and clinical findings suggestive of systemic citrate accumulation during RCA. Abbreviations: RCA, regional citrate anticoagulation; T/iCa, total-to-ionized calcium ratio.

**Table 4:** Recommended monitoring parameters during regional citrate anticoagulation for continuous kidney replacement therapy (RCA-CKRT). Recommendations are based on the authors’ synthesis of published RCA protocols and expert consensus recommendations [7,8,9,22,23,24,31,32]. Proposed thresholds are intended to guide clinical decision-making and have not been prospectively validated.

Parameter	Normal Range	Critical Threshold	Monitoring Frequency
iCa (mmol/L)	1.1–1.3	<0.8	Every 4–6 h
T/iCa ratio	1.8–2.2	>2.5	Every 4–6 h
Arterial blood gas (pH/PCO_2_)	7.35–7.45	<7.30 or clinically significant hypercapnia	Every 4–6 h (more frequently in hypercapnic patients)
Anion gap (mmol/L)	8–16	>20	Every 4–6 h
Lactate (mmol/L)	<2.0	>4.0	Every 6–12 h
iMg (mmol/L)	0.5–0.7	<0.4	Daily
Sodium (mmol/L)	135–145	>150	Every 6–12 h

Legend. Suggested monitoring parameters during RCA-CKRT; frequency should be individualized according to clinical stability and metabolic risk [7,8,23,24]. Abbreviations: CKRT, continuous kidney replacement therapy; iCa, ionized calcium; iMg, ionized magnesium; PCO_2_, partial pressure of carbon dioxide; RCA, regional citrate anticoagulation; T/iCa, total-to-ionized calcium ratio.

## Data Availability

No new data were created or analyzed in this study. Data sharing is not applicable to this article.
